# Circulating miR-10b-5p as a candidate biomarker of atrial fibrillation recurrence after catheter ablation: a two-phase translational study

**DOI:** 10.1093/europace/euag097

**Published:** 2026-04-28

**Authors:** Εmmanouil P Vardas, Evangelos Oikonomou, Stylianos Tzeis, Panagiotis Theofilis, Maria Gazouli, Eleni Papadogeorgaki, Konstantinos Belogiannis, Dimitrios Asvestas, Emmanouil Vavouris, Dimitrios Charitos, Dimitris Tousoulis

**Affiliations:** Department of Cardiac Electrophysiology, Barts Heart Centre, St Bartholomew's Hospital, West Smithfield, London EC1A 7BE, UK; 3rd Department of Cardiology, ‘Sotiria’ Chest Diseases Hospital, Medical School, National and Kapodistrian University of Athens, Athens 11527, Greece; Department of Cardiology, Mitera Hospital, Athens, Greece; 1st Department of Cardiology, ‘Hippokration’ General Hospital, National and Kapodistrian University of Athens, School of Medicine, Athens, Greece; Laboratory of Biology, Department of Basic Medical Sciences, Medical School, National and Kapodistrian University of Athens, Athens 11527, Greece; Central Diagnostic Laboratories, Hygeia Hospital Athens, Athens 15123, Greece; Central Diagnostic Laboratories, Hygeia Hospital Athens, Athens 15123, Greece; Department of Cardiology, Mitera Hospital, Athens, Greece; Department of Cardiology, Mitera Hospital, Athens, Greece; Department of Cardiology, Mitera Hospital, Athens, Greece; 1st Department of Cardiology, ‘Hippokration’ General Hospital, National and Kapodistrian University of Athens, School of Medicine, Athens, Greece

**Keywords:** Atrial fibrillation, Catheter ablation, Recurrence, MicroRNA, Biomarkers, miR-10b-5p

## Abstract

**Aims:**

The identification of reliable biomarkers for atrial fibrillation (AF) recurrence post-catheter ablation remains a clinical challenge. This study aimed to identify circulating microRNAs (miRNAs) associated with post-ablation AF recurrence and examine their underlying molecular pathways using an integrative translational approach.

**Methods and results:**

This two-phase study included a discovery case-control phase (*n* = 29) followed by a prospective validation cohort (*n* = 126). In the discovery phase, 84 miRNAs were quantified via real-time PCR, and candidates were selected using differential expression analysis and machine learning. In the validation phase, five candidate miRNAs (hsa-miR-342-3p, hsa-miR-424-5p, hsa-miR-486-5p, hsa-miR-10b-5p, and hsa-let-7d-5p) were further evaluated to assess their prognostic performance. Pathway enrichment analysis was performed for the most predictive miRNA. Differential expression analysis identified 14 miRNAs to be significantly associated with AF recurrence. In the validation cohort, hsa-miR-10b-5p showed the highest discriminative performance (AUC = 0.96, *P* < 0.001). Multivariable logistic regression confirmed that lower expression of hsa-miR-10b-5p was an independent predictor of recurrence (OR = 0.06, *P* < 0.001), significantly improving the baseline clinical model (ΔR = 63.3%). Pathway analysis linked hsa-miR-10b-5p to FOXO signalling, p53 signalling, circadian rhythm and cellular senescence, pathophysiologic mechanisms that are critical to atrial remodelling, and fibrotic persistence.

**Conclusion:**

Down-regulation of circulating hsa-miR-10b-5p was independently associated with AF recurrence after catheter ablation and improved risk discrimination beyond clinical variables. These findings support its potential role as a prognostic biomarker, although further multicentre validation is required before clinical application.

## Graphical Abstract

**Graphical Abstract:**
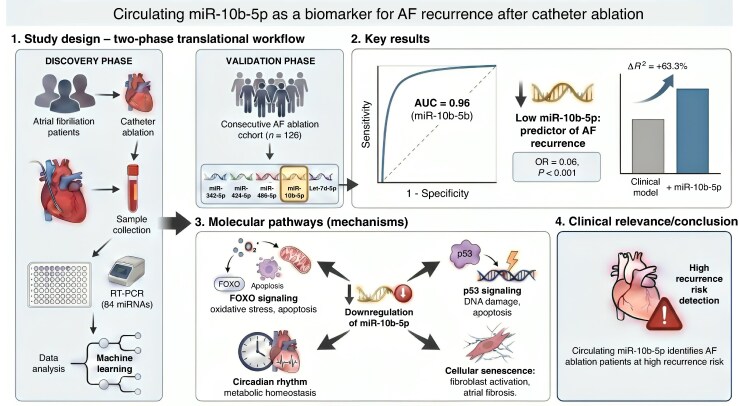

What’s new?

- Down-regulation of circulating hsa-miR-10b-5p levels were independently associated with atrial fibrillation recurrence after catheter ablation and improved risk prediction beyond clinical variables.

## Introduction

Over the past decades, significant progress has been made in our epidemiological understanding of atrial fibrillation (AF) prevalence,^1,2^ as well as in the study of its pathophysiology and therapeutic management,^3,4^ largely driven by emerging technologies that enable more precise and effective interventions.^5^

In the ongoing search for biomarkers associated with the development and recurrence of AF after catheter ablation, recent research has increasingly focused on the role of microRNAs (miRNAs)—pathophysiological factors linked to arrhythmia initiation and progression^6,7^—as potential prognostic indicators of ablation outcomes.^8,9^

MiRNAs are small non-coding RNAs of approximately 18–25 nucleotides in length that regulate post-transcriptional gene expression.^10^ They are known to be involved in several key processes related to AF initiation and recurrence, including fibrosis, inflammation, and oxidative stress.^11–13^ These molecules are remarkably stable and tissue-specific, allowing their reliable detection in peripheral blood.^14^

In recent years, a substantial number of studies have explored this field, highlighting the potential role of various non-coding RNAs in AF pathophysiology.^15^ However, while some findings consistently support the aetiopathogenic relevance of specific miRNAs, others have led to contradictory or inconclusive results, underscoring the complexity of AF biology.^16,17^

The aim of the present study was therefore to identify circulating miRNAs associated with post-ablation AF recurrence, using a two-phase design comprising a discovery case-control phase and a prospective validation cohort. An integrative statistical and machine learning approach was applied to define specific miRNAs predictive of AF recurrence and to elucidate their underlying molecular pathways.^18,19^ Ultimately, the study seeks to link molecular expression data with clinical outcomes, providing a mechanistic understanding of AF recurrence and supporting the development of biomarker-based tools for personalized care.

## Material and methods

### Study design, population, and procedure characteristics

This was a single-centre, two-phase study consisting of a case-control discovery phase and an independent prospective validation cohort (see Supplementary material online, *Figure S1*). The source population consisted of all consecutive patients undergoing catheter ablation for paroxysmal or persistent AF at our institution during the study period. Inclusion criteria included age ≥18 years, catheter ablation for symptomatic paroxysmal or persistent AF, and availability of clinical follow-up for at least 12 months. Exclusion criteria included age <18 years, pregnancy, chronic kidney disease, severe liver disease, coronary artery disease, and active malignancy, in accordance with standard eligibility criteria.^20^ Sample size was determined based on expected miRNA effect sizes observed in previous studies of AF recurrence, targeting a power of 80% and α = 0.05.^21,22^

All subjects underwent either point-by-point irrigated radiofrequency ablation (SmartTouch SF, Biosense Webster) or pulsed field ablation (PFA) using either a large-footprint lattice-tip catheter system (Affera, Medtronic) or a single-shot system (Farapulse, Boston Scientific) for pulmonary vein isolation.^5,23,24^ Additional targeted ablation was performed at the operator’s discretion, reflecting real-world procedural variability.

Oral anticoagulation was maintained for at least 3 months following the procedure and continued lifelong in patients with a CHA_2_DS_2_-VASc score ≥1 in men or ≥2 in women.^25,26^ Antiarrhythmic therapy was discontinued after 3 months, consistent with consensus guidelines on blanking period management.^27^

Each patient underwent serial 48 h Holter monitoring at 3, 6, and 12 months following the procedure. This approach aligns with standard research protocols, though brief monitoring may underestimate recurrence compared with continuous surveillance.^28,29^ Additionally, all symptomatic patients were advised to obtain ECG recordings to confirm possible recurrence. Recurrence was defined as any documented episode of AF or atrial tachycardia lasting more than 30 s after a 3 month blanking period.^25,30,31^

The study was conducted in accordance with the Declaration of Helsinki and approved by the institutional ethics committee.^32^ All participants provided written informed consent.

### Discovery phase (case-control study)

In the discovery phase, a nested case-control study was conducted including 29 patients who underwent catheter ablation for AF. Fifteen patients who experienced AF recurrence during a 12 month follow-up (cases) and 14 who remained arrhythmia-free (controls) were selected. The discovery cohort was derived from a prospective registry of consecutive patients undergoing catheter ablation for AF at our centre. All ablated patients were followed according to the standardized protocol described above. From this source population, patients were eligible for biomarker analysis if a pre-ablation plasma sample of adequate quality was available and complete 12 month follow-up had been obtained.

All eligible patients with documented recurrence were first identified. For each recurrence case, one control patient without recurrence was then selected from the same source cohort with the aim of achieving comparability in key baseline clinical characteristics, including age, sex, body mass index, hypertension, diabetes mellitus, dyslipidaemia, left atrial volume index, and AF type (paroxysmal vs. persistent), thereby reducing confounding (see Supplementary material online, *Table S1*).

Patient selection for miRNA measurement was completed prior to laboratory analysis, and investigators performing miRNA quantification were blinded to clinical outcomes.

A panel of 84 preselected miRNAs was quantified using real-time PCR. The miRNAs were selected a priori from a targeted panel enriched for candidates previously associated with AF pathophysiology and post-ablation recurrence in prior human and experimental studies.^10–16^ Differential expression analysis, integrating fold regulation and adjusted *P*-values, was complemented by a decision tree-based machine learning approach to identify the miRNAs most strongly associated with AF recurrence. Machine learning methods have shown particular value in high-dimensional miRNA biomarker discovery and were therefore employed to enhance signal detection beyond conventional statistical testing.^33^

Given the limited sample size and exploratory objective, this discovery phase was designed to identify promising candidate miRNAs for validation.

### Validation phase (prospective cohort study)

In the validation phase, candidate miRNAs identified during the discovery stage were measured in an independent prospective cohort of 126 consecutive patients undergoing AF ablation at the same centre after completion of the discovery phase.

Five candidate miRNAs, hsa-miR-342-3p, hsa-miR-424-5p, hsa-miR-486-5p, hsa-miR-10b-5p, and hsa-let-7d-5p, were advanced based on discovery-phase patterns and their biological plausibility.^34–36^

Follow-up procedures mirrored the discovery-phase protocol, including 48 h Holter monitoring and ECG documentation of symptomatic events. Recurrence definitions followed consensus standards.

### Blood samples collection and microRNA quantification

Peripheral venous blood samples were obtained via the femoral venous sheath immediately before ablation, in accordance with standard preprocedural biomarker collection protocols. Samples were collected into EDTA tubes, centrifuged at 1000 x g at 4°C for 10 min to separate plasma, and the resulting plasma aliquots were stored at −80°C until analysis to preserve miRNA integrity.^37,38^

MiRNAs were isolated from plasma using an extraction kit according to the manufacturer’s instructions, and miRNA quality was assessed by spectrophotometry. Additionally, reverse transcription was performed using the miRCURY LNA RT Kit (QIAGEN), a validated platform for circulating miRNA profiling in plasma and other biofluids.^39^

Relative miRNA expression was calculated using the 2^−ΔΔCt method. Ct values were normalized to the geometric mean of the two most stable endogenous reference RNAs, SNORD38B and SNORD44, identified by stability analysis using the geNorm algorithm across the entire dataset (see supplementary material online, *F*igure  *S2*).^40,41^

Differential expression between the AF recurrence and non-recurrence groups was evaluated using unpaired Student’s *t*-tests or Mann–Whitney *U* tests, according to the normality of distribution which was evaluated with the Shapiro–Wilk test. The resulting *P*-values were adjusted for multiple testing using the Benjamini–Hochberg procedure to control the false discovery rate (FDR).^42^

### Machine learning-based feature selection

In the present study, machine learning methods were used exclusively as an exploratory feature-selection approach during the biomarker discovery phase to identify the most informative miRNAs from a high-dimensional expression dataset. The resulting candidate miRNAs were subsequently evaluated using conventional statistical models in an independent validation cohort.

The relative expression of 84 miRNAs was assessed to identify those most strongly associated with AF recurrence and to select candidate miRNAs for subsequent validation in the remaining study population.

To explore expression patterns and visualize miRNAs with the greatest discriminatory capacity between patients with and without AF recurrence, a heatmap was generated based on the most informative features.^43,44^

Discriminative miRNAs were further identified using a decision tree classifier implemented with the *rpart* package in R (version 4.1.2). Prior to model training, miRNA cycle threshold (Ct) values were standardized using z-score normalization. MiRNAs exhibiting low variance across samples (variance ≤ 1.0) were excluded to minimize noise and enhance model generalizability.^45–47^

Model performance was evaluated using leave-one-out cross-validation (LOOCV) applied to the filtered and normalized dataset, allowing assessment of the predictive ability of miRNA expression profiles for AF recurrence.^48^

Reporting of the machine learning component of this study follows the recommendations of the European Heart Rhythm Association (EHRA) State-of-the-Art document on Artificial Intelligence in Clinical Electrophysiology.^49^

### Statistical analysis

As with the discovery, continuous variables were first assessed for normality using the Shapiro–Wilk test. Depending on the distribution, group comparisons were performed using either Student’s *t*-test or the Mann–Whitney *U* test and are presented as mean ± standard deviation or median (interquartile range). Categorical variables are shown as frequencies and percentages and were compared using χ^2^ or Fisher’s exact tests.^50,51^

In the validation phase, the five miRNAs with the strongest differential expression were further evaluated. Associations between log_two-fold_-change miRNA expression and AF recurrence were examined using multivariable logistic regression adjusted for established clinical predictors (age, sex, BMI, hypertension, dyslipidaemia, diabetes mellitus, LAVi, and paroxysmal AF status).^52^

The incremental predictive value of individual miRNAs was assessed using hierarchical logistic regression. For each multivariable model, predicted probabilities were derived from the fitted regression models, and model discrimination was evaluated using receiver operating characteristic (ROC) curve analysis.^53^ Pairwise comparisons of area under the curves (AUCs) were performed using the DeLong test, and differences in AUCs (ΔAUC) were reported.

Statistical analyses were performed using SPSS (version 29.0) and R (version 4.1.2). All tests were two-sided, with *P* < 0.05 considered statistically significant.

### miRNA pathway analysis

Based on differential expression analyses, hsa-miR-10b-5p, the most strongly associated miRNA with AF recurrence, was selected for pathway enrichment analysis to explore potential underlying molecular mechanisms.

Initially, functional pathway analysis was performed using DIANA-miRPath v4.0 (http://62.217.122.229:3838/app/miRPathv4), applying Pathway Union mode and experimentally validated gene targets retrieved from TarBase v8.0. Enrichment was assessed against KEGG pathways using Fisher’s exact test with FDR correction, with FDR < 0.05 considered statistically significant.^54,55^

In order to prioritize biologically relevant mechanisms related to hsa-miR-10b-5p, the pathways with the lowest FDR values and the highest number of experimentally validated target genes were selected. Finally, visualization of miR-10b-5p target genes within enriched KEGG pathways was performed using Pathview.

## Results

### Exploratory results

**Figure 1:**
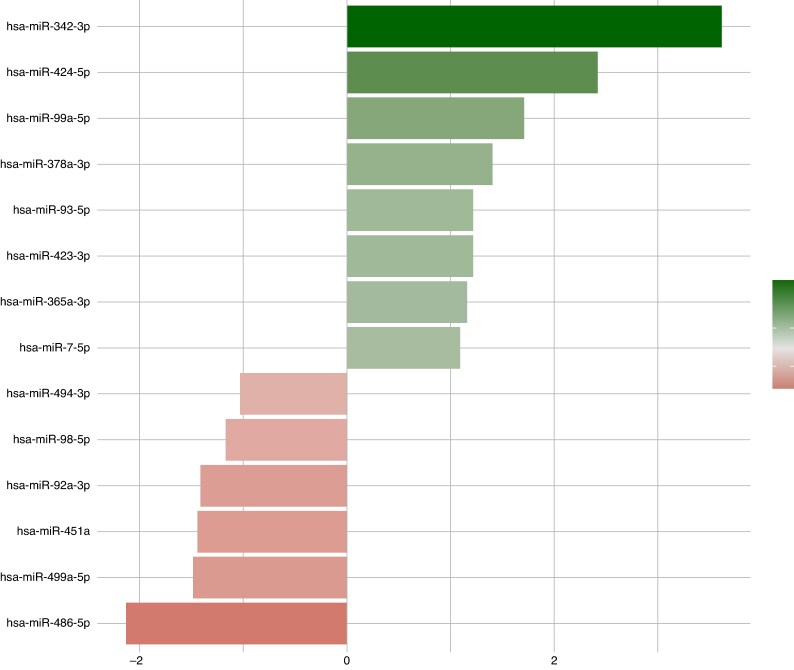
Bar plot of top differentially expressed miRNAs between Rec and Nrec groups. This plot displays the log2 fold change in mean expression for selected miRNAs comparing Recurrence (Rec) vs. Non-recurrence (Nrec) sample groups. Upregulated miRNAs are shown on the right (positive values), while downregulated miRNAs are shown on the left (negative values) in patients with Rec. Among the studied miRNAs, miR-342-3p, miR-424-5p, and miR-99a-5p were the most up-regulated, whereas miR-486-5p, miR-499a-5p, and miR-451a were the most down-regulated in the Rec group.

In the exploratory analysis, 29 patients were included, comprising 15 patients with AF recurrence and 14 without recurrence. Baseline clinical and demographic characteristics are presented in Supplementary material online, *Table S1*. Expression levels and fold regulation of all 84 analysed miRNAs according to AF recurrence status are shown in Supplementary material online, *Table S2*. Exploratory univariable comparisons across the miRNAs identified 14 candidates showing nominal associations with AF recurrence (*P* < 0.05; *Figure 1*). Since none of the miRNAs reached the conventional FDR significance threshold, these findings were interpreted as hypothesis-generating and were used to prioritize candidate miRNAs for subsequent validation in the independent cohort.

**Figure 2:**
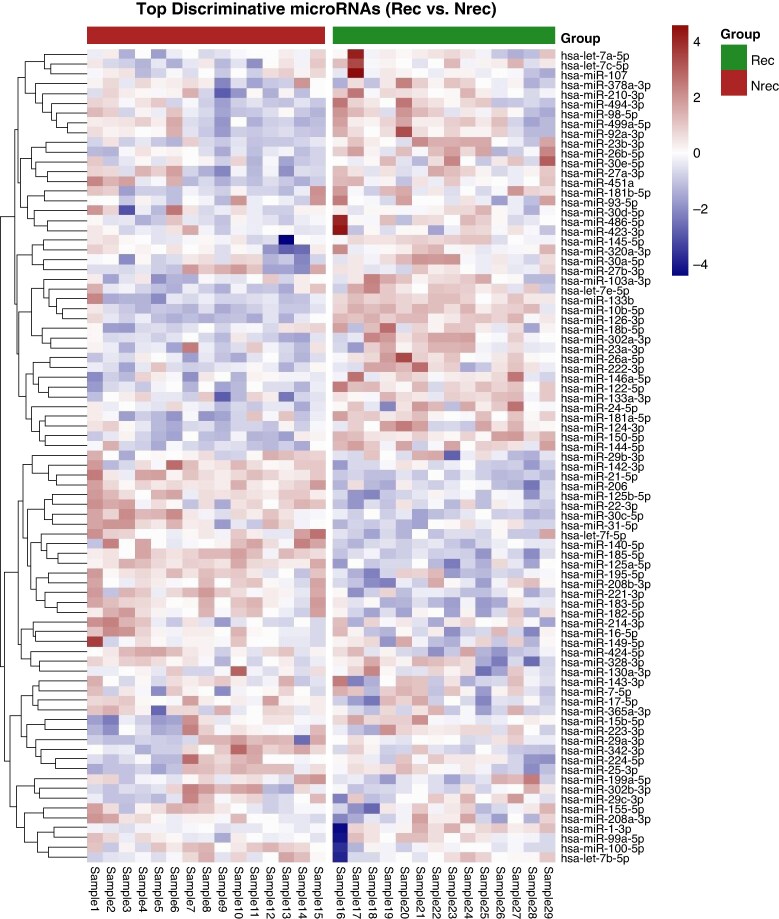
Heatmap of most dysregulated miRNAs differentiating AF recurrence. Expression values were row-wise z-scored and plotted using hierarchical clustering (Euclidean distance, complete linkage) for miRNAs (rows), while samples (columns) were grouped by condition (recurrence, Rec vs. Non-recurrence, Nrec).

**Figure 3:**
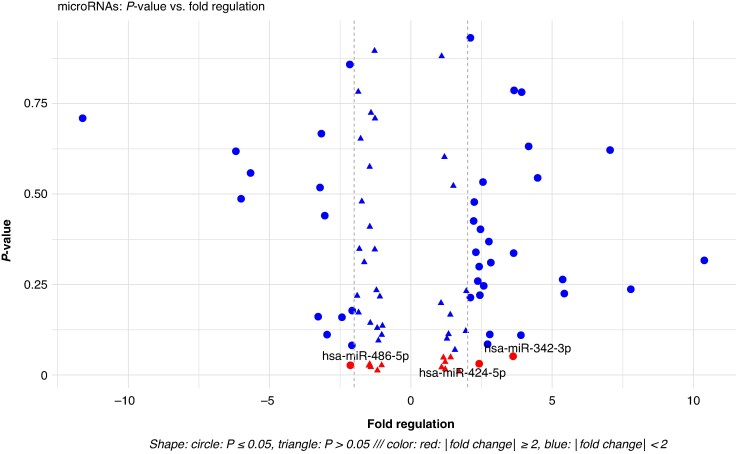
Differential expression of candidate miRNAs. Individual miRNAs are shown as single points, x-axis consists of the fold regulation of each miRNA, while the y-axis shows statistical significance (*P*-value). Circle, |fold change| ≥ 2; triangle, |fold change| < 2; colour indicates *P*-value; red, *P* ≤ 0.05 (statistically significant); blue, *P* > 0.05 (not significant). Notably, hsa-miR-342-3p, hsa-miR-424-5p, and hsa-miR-486-5p appear as prominent candidates, combining high fold regulation with statistical significance.

A heatmap (*Figure 2*) depicting the expression profiles of the 84 analysed miRNAs with the highest absolute log_two-fold_ change was associated with clear separation between patients with and without AF recurrence. Among the differentially expressed miRNAs, hsa-miR-342-3p and hsa-miR-424-5p were up-regulated, whereas hsa-miR-486-5p was down-regulated in the recurrence group, each showing at least a two-fold difference between groups (*Figure 3*).^56^

**Figure 4:**
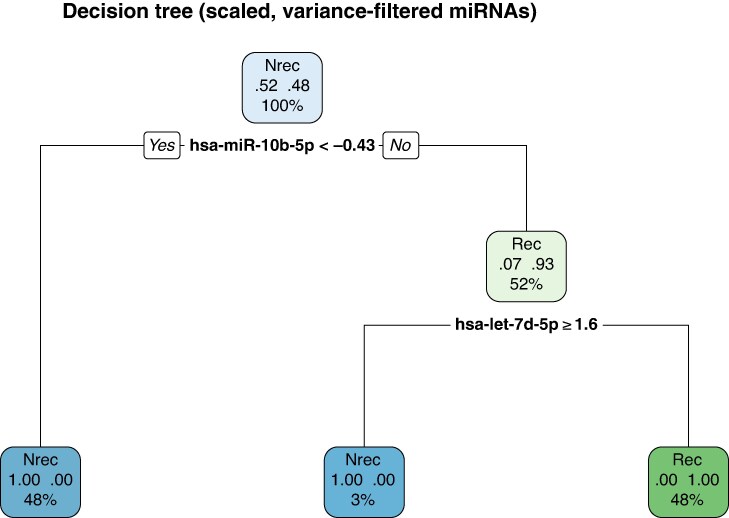
Decision tree classifier based on normalized expression levels of circulating miRNAs for the prediction of AF recurrence following ablation. The model was trained on z-score scaled Ct values after filtering for low-variance miRNAs. The tree shows optimal splits on hsa-miR-10b-5p and hsa-let-7d-5p, resulting in complete class separation between recurrence (Rec) and non-recurrence (Nrec) patients. Node labels show class probabilities and sample percentages within each leaf.

Decision tree analysis revealed that hsa-miR-10b-5p and hsa-let-7d-5p were the most informative features in distinguishing patients with recurrent vs. non-recurrent AF following ablation (*Figure 4*).^57,58^ The initial split of the tree was based on hsa-miR-10b-5p expression, with lower z-score values (< −0.43) associated with the non-recurrence group. Among patients with higher hsa-miR-10b-5p levels, further stratification was achieved by hsa-let-7d-5p. Patients with hsa-let-7d-5p expression ≥ 1.6 were classified exclusively to the recurrence group, which represented 48% of the total cohort. The model showed clear separation between recurrence and non-recurrence groups with minimal misclassification.

### Prognostic performance of miRNAs

Based on discovery-phase findings, five miRNAs (hsa-miR-342-3p, hsa-miR-424-5p, hsa-miR-486-5p, hsa-miR-10b-5p, and hsa-let-7d-5p) were selected for validation in a prospective cohort of 126 consecutive patients undergoing AF ablation. Baseline clinical and demographic characteristics are shown in *Table 1*. Among the 126 patients included in the validation cohort, 29 patients (23.0%) experienced AF recurrence during follow-up. Recurrence rates were comparable across ablation modalities (radiofrequency 21.3%, PFA—Farapulse 26.3%, PFA—Affera 22.2%; *Table 1*), with no significant association between energy source and AF recurrence.

**Table 1:** Baseline characteristics of the validation cohort

Variable	Overall	AF recurrence (*n* = 29)	No AF recurrence (*n* = 97)	*P*-value
Age, years	62.3 (10.4)	61.9 (10.9)	62.5 (10.3)	0.79
Male sex, *n* (%)	94 (74.6)	19 (65.5)	75 (77.3)	0.20
BMI, kg/m^2^	28.6 (5.6)	28.9 (6.3)	28.5 (5.3)	0.77
Hypertension, *n* (%)	64 (50.8)	13 (44.8)	51 (52.6)	0.46
Diabetes mellitus, *n* (%)	7 (5.6)	4 (13.8)	3 (3.1)	0.03
Dyslipidaemia, *n* (%)	47 (37.3)	9 (31)	38 (39.2)	0.43
Thyroid disease, *n* (%)	13 (10.3)	3 (10.3)	10 (10.3)	1.00
Coronary artery disease, *n* (%)	8 (6.3)	2 (6.9)	6 (6.2)	0.89
Paroxysmal, *n* (%)	80 (63.5)	16 (55.2)	64 (66)	0.29
Persistent, *n* (%)	46 (36.5)	13 (44.8)	33 (34)	
RFA, *n* (%)	61 (48.4)	13 (44.8)	48 (49.5)	
PFA—Farapulse, *n* (%)	38 (30.2)	10 (34.5)	28 (28.9)	
PFA—Affera, *n*	27 (21.4)	6 (20.7)	21 (21.6)	
AF recurrence, *n* (%)	29 (23)			
LAVi, mL/m^2^	32.4 (26.1, 39.6)	30.2 (23, 36.7)	33.1 (27.7, 40.8)	0.11
LA diameter, mm	37 (35, 42)	38 (35, 40)	37 (36, 42)	0.92

**Figure 5:**
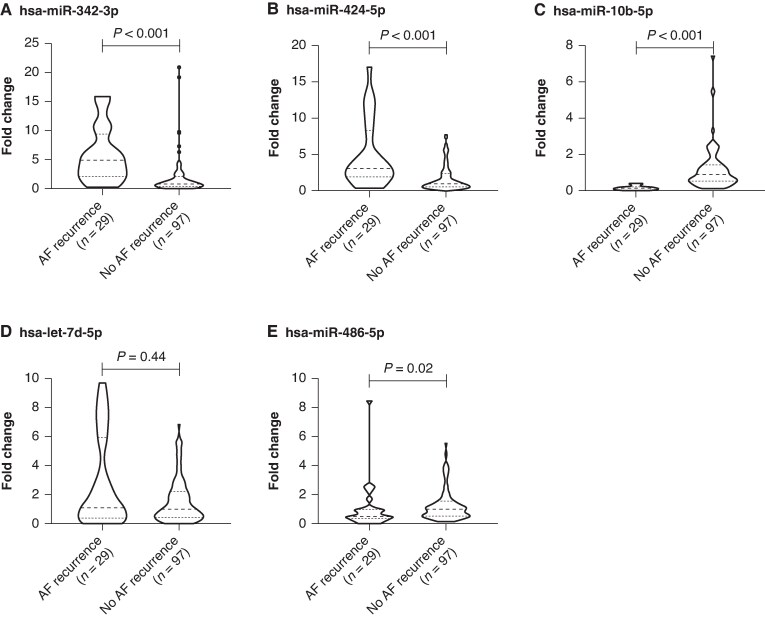
Violin plots of circulating miRNA fold-changes by AF recurrence status. Bars show mean fold change (relative expression) ± SEM in patients with AF recurrence (black) vs. No AF recurrence (grey). *P*-values are from two-sided between-group tests. (*A*) hsa-miR-342-3p: higher in AF recurrence (*P* < 0.001). (*B*) hsa-miR-424-5p: higher in AF recurrence (*P* < 0.001). (*C*) hsa-miR-10b-5p: lower in AF recurrence (*P* < 0.001). (*D*) hsa-let-7d-5p: similar between groups (*P* = 0.44). (*E*) hsa-miR-486-5p: lower in AF recurrence (*P* = 0.02).

In the validation cohort, consisting of 126 consecutive patients, AF recurrence was associated with differential expression of hsa-miR-342-3p, hsa-miR-424-5p, hsa-miR-10b-5p, and hsa-miR-486-5p, whereas hsa-let-7d-5p did not differ between recurrence and non-recurrence groups (*Figure 5*).

**Figure 6:**
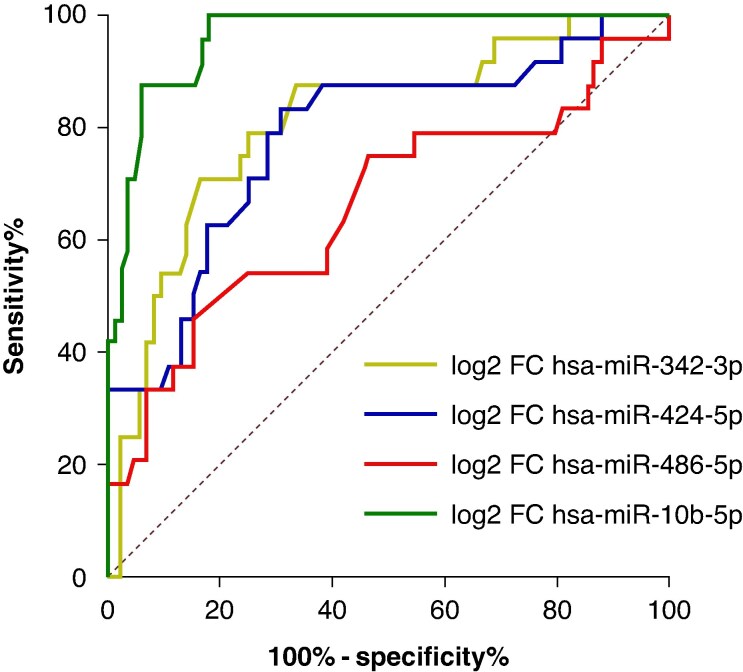
ROC curves of candidate miRNAs. ROC curves depicting the diagnostic performance of hsa-miR-342-3p (yellow), hsa-miR-424-5p (blue), hsa-miR-486-5p (red), and hsa-miR-10b-5p (green). Among these, hsa-miR-10b-5p showed the highest discriminative ability, with an AUC of 0.96 (*P* < 0.001).

ROC curve analysis showed the highest discriminative performance for hsa-miR-10b-5p, followed by hsa-miR-342-3p and hsa-miR-424-5p, while hsa-miR-486-5p showed limited discrimination (*Figure 6*). Pairwise AUC comparisons confirmed the superiority of hsa-miR-10b-5p over hsa-miR-486-5p (ΔAUC = 0.267, *P* < 0.001), hsa-miR-342-3p (ΔAUC = 0.751, *P* < 0.001), and hsa-miR-424-5p (ΔAUC = 0.721, *P* < 0.001) (see Supplementary material online, *Table S3*).

In multivariable logistic regression models adjusted for clinical and echocardiographic covariates, higher expression of hsa-miR-342-3p and hsa-miR-424-5p and lower expression of hsa-miR-10b-5p were independently associated with AF recurrence (*Table 2*). Addition of each miRNA individually to a baseline clinical model significantly improved model fit (see Supplementary material online, *Table S4*), with the largest improvement observed for hsa-miR-10b-5p (Δχ^2^ = 50.9, *P* < 0.001; ΔR^2^ = 63.3%). A model including all five miRNAs achieved the highest overall fit (Δχ^2^ = 52.5, *P* < 0.001; ΔR^2^ = 63.5%).

**Table 2:** Multivariable regression analysis of the association between the studied miRs and AF recurrence post-catheter ablation

Variable	OR	95% CI	*P*-value
Log2 FC hsa-miR-342-3p	2.16	1.35–3.46	0.001
Log2 FC hsa-miR-424-5p	1.85	1.13–3.01	0.01
Log2 FC hsa-miR-10b-5p	0.06	0.01–0.33	<0.001
Log2 FC hsa-miR-486-5p	0.64	0.38–1.07	0.09

Adjusted for male sex, age, BMI, hypertension, dyslipidaemia, diabetes mellitus, left atrial volume index, and paroxysmal AF status.

FC: fold change.

ROC analyses based on predicted probabilities from the multivariable models were associated with high discriminatory performance across models (see Supplementary material online, *Table S5*). Addition of hsa-miR-10b-5p to clinical and echocardiographic variables significantly improved discrimination (see Supplementary material online, *Figure S3*), whereas inclusion of all five miRNAs did not confer further benefit (ΔAUC = 0.007, *P* = 0.29)

### Pathway analysis

**Figure 7:**
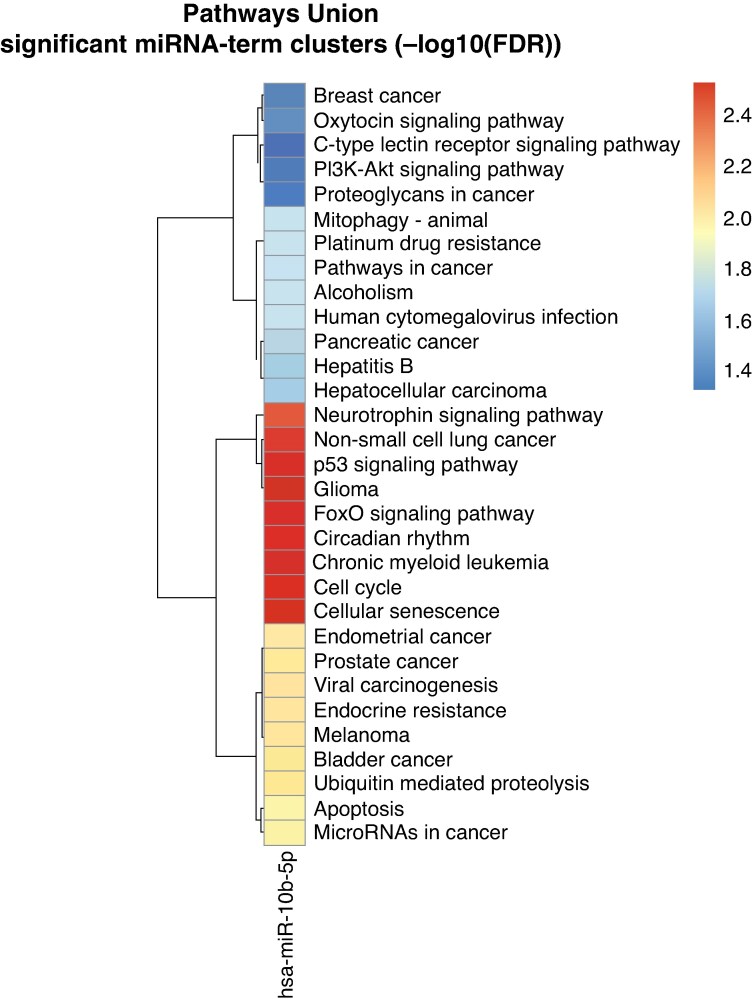
Pathway enrichment heatmap of hsa-miR-10b-5p. Hierarchical clustering heatmap showing significantly enriched KEGG pathways targeted by hsa-miR-10b-5p, as analysed using DIANA-miRPath v4.0 (pathways union mode). The colour scale represents the -log_10_(FDR) values, with deeper red indicating higher statistical significance of enrichment.

Pathway enrichment analysis identified multiple KEGG pathways significantly associated with hsa-miR-10b-5p (FDR < 0.05), with the strongest enrichment observed in FOXO signalling, p53 signalling, cell–cycle/senescence, and circadian rhythm-related pathways (*Figure 7*). These pathways have been previously implicated in key biological processes relevant to AF, including oxidative stress regulation, apoptosis, DNA damage response, and fibrotic remodelling.^59-61^

The FOXO network, which includes FOXO3, PTEN, PDK1, and BCL2L1, mediates oxidative stress resistance, apoptosis suppression, and metabolic adaptation, all central to atrial structural remodelling (see Supplementary material online, *Figure S4*).^61^

The p53 pathway (TP53, MDM2, CDKN1A, BCL2L1) is a key regulator of DNA damage response, cell–cycle arrest, senescence, and apoptosis, with multiple studies linking it to remodelling and arrhythmogenic vulnerability (see Supplementary material online, *Figure S5*).^62^

Cell–cycle/senescence pathways involving CDKN1A, CDKN2A, and CCND2 highlight fibroblast activation and extracellular matrix turnover—core mechanisms in AF substrate persistence (see Supplementary material online, *Figure S6*–S7).^63^

Circadian rhythm pathways involving RORA, NR1D1, and CREB1 integrate electrophysiological timing, metabolic homeostasis, and autonomic influences, offering a mechanistic link between circadian disruption and AF susceptibility (see Supplementary material online, *Figure S8*).^64^

Overall, these enrichment results provide mechanistic coherence, integrating miRNA dysregulation with known biological processes underlying AF recurrence.

## Discussion

In this two-phase study, clinical and molecular data were integrated to explore the association between circulating miRNAs and AF recurrence following treatment with catheter ablation. The analysis of the data revealed several noteworthy observations that, on the one hand, contribute to our understanding of the mechanisms underlying AF substrate and, on the other hand, underscore the role of miRNA regulation on gene expression and protein synthesis in AF pathogenesis.

First, we show that differential expression of several miRNAs, including both up-regulated and down-regulated profiles, is associated with AF recurrence after catheter ablation. Moreover, the application of machine learning approaches allowed the identification, from a large set of miRNAs, of those most strongly predicted AF recurrence including hsa-miR-342-3p, hsa-miR-424-5p, hsa-miR-486-5p, hsa-miR-10b-5p, and hsa-let-7d-5p. Importantly, internal validation in an independent and larger cohort of patients undergoing AF catheter ablation confirmed the predictive performance of most identified miRNAs in the exploratory cohort, with hsa-miR-10b-5p showing the strongest predictive ability.

To date, the mechanisms causing the genesis and recurrence of AF have primarily been interpreted through clinical risk factors (e.g. age, hypertension, ischaemic heart disease) and pathological evidence, such as left atrial dilation, atrial fibrosis, and atrial scarring.^65^ These alterations are increasingly conceptualized within the broader framework of atrial cardiomyopathy, defined as a spectrum of structural, architectural, contractile, and electrophysiological changes affecting the atria that may lead to clinically relevant manifestations such as AF and thromboembolism.^66^ However, it remains unknown whether these risk features are simply associated with AF or play a causal pathophysiologic role. Moreover, the predictive performance of current clinical models integrating these variables remains modest. Based on our findings, standardized measurement of circulating miRNAs may improve prognostic accuracy for post-ablation AF recurrence beyond established clinical models.

The analytic framework of this study, combining differential expression analysis with machine learning-based classification and mechanistic pathway exploration, aligns with contemporary biomarker development strategies. The integration of statistically robust feature selection with interpretable machine learning enhanced the biological coherence and the predictive ability of clinical based models. The significant improvement of a clinical based model following the inclusion of hsa-miR-10b-5p supports its potential role as a biologically plausible and mechanistically relevant biomarker for AF recurrence after catheter ablation.

To provide biological context for these prognostic associations, *in silico* pathway enrichment analysis identified four KEGG pathways enriched for experimentally validated hsa-miR-10b-5p targets: FOXO signalling, p53 signalling, cellular senescence, and circadian rhythm. These pathways have previously been implicated in oxidative stress regulation, DNA damage response, fibroblast activation, and electrophysiological remodelling—processes broadly consistent with the known biology of AF substrate.^67–72^ It is important to emphasise, however, that these associations are derived entirely from *in silico* integration of target gene databases and do not establish a causal or mechanistic role for hsa-miR-10b-5p in AF recurrence. They should therefore be interpreted as hypothesis-generating observations that may inform the design of future experimental studies rather than as mechanistic conclusions supported by the present data.

## Limitations

Despite the coherence of these results, several limitations warrant careful consideration.

This study was conducted at a single centre with a modest sample size, particularly during the discovery phase, which increases the risk of overfitting. The single-centre design may limit the generalizability of these findings to other populations and clinical settings.

Different ablation technologies were used. Although recurrence rates were similar across modalities, procedural heterogeneity may still represent a source of residual confounding.^73–75^

Additionally, although we adjusted for major clinical covariates, residual confounding cannot be excluded, and we cannot conclude whether miR-10b-5p acts as a biomarker, a bystander, or an active modulator of atrial remodelling.

Finally, although FDR correction was applied, high-dimensional miRNA analyses inherently carry a risk of false-positive associations.^76^

## Conclusion

This exploratory two-phase study suggests that altered circulating miRNA expression profiles are associated with AF recurrence post-ablation. Particularly the down-regulation of circulating hsa-miR-10b-5p was independently associated with AF recurrence after catheter ablation and improved prediction beyond clinical variables. Larger multicentre, longitudinal, and mechanistically integrated studies—including tissue-level profiling, serial sampling, and experimental validation—are necessary to confirm the prognostic utility of hsa-miR-10b-5p and to determine its clinical utility.

## Supplementary Material

euag097_Supplementary_Data

## Data Availability

The data that support the findings of this study will be shared on reasonable request to the corresponding author.
