# Supplementary material for: Circulating miR-10b-5p as a candidate biomarker of atrial fibrillation recurrence after catheter ablation: a two-phase translational study
Source: Europace. 2026 Apr 28;28(5):euag097. doi: 10.1093/europace/euag097 (PMC13179786; doi:10.1093/europace/euag097)
Supplement: euag097_Supplementary_Data [file euag097_supplementary_data.zip › Supplementary material 2.docx]

**Contents**

[**Supplementary Table 1. Baseline characteristics of the exploratory phase case-control group.** 2](#_Toc211714553)

[**Supplementary Table 2. Fold regulations. p-values. and FDRs of all 84 miRNAs.** 3](#_Toc211714554)

[**Supplementary Table 3. Comparative AUROC values for each miR in predicting AF recurrence after catheter ablation.** 3](#_Toc211714555)

[**Supplementary Table 4. Improvement in model fit after inclusion of individual and combined miRs to the baseline clinical model for predicting AF recurrence after catheter ablation.** 3](#_Toc211714556)

[**Supplementary Table 5. ROC Curve Analysis of Predicted Probabilities from Logistic Regression Models Based on log2 Fold Change of Candidate miRs.** 6](#_Toc211714557)

[**Supplementary Figure 1. Stepwise analytical workflow for the identification and validation of circulating miRs associated with AF recurrence after ablation.** 8](#_Toc211714558)

[**Supplementary Figure 2. ROC curves comparing predictive models for AF recurrence after ablation.** 9](#_Toc211714559)

[**Supplementary Figure 3. FOXO signaling pathway targeted by hsa-miR-10b-5p.** 11](#_Toc211714560)

[**Supplementary Figure 4. p53 signaling pathway targeted by hsa-miR-10b-5p.** 12](#_Toc211714561)

[**Supplementary Figure 5. Cell cycle pathway targeted by hsa-miR-10b-5p.** 13](#_Toc211714562)

[**Supplementary Figure 6. Cellular senescence pathway targeted by hsa-miR-10b-5p.** 14](#_Toc211714563)

[**Supplementary Figure 7. Circadian rhythm pathway targeted by hsa-miR-10b-5p.** 15](#_Toc211714564)

# **Supplementary Table 1. Baseline characteristics of the exploratory phase case-control group.**

| Variable | Overall | AF recurrence (n=15) | No AF recurrence (n=14) | P-value |
| --- | --- | --- | --- | --- |
| Age, years | 62.1 (10.5) | 60 (9.5) | 64.2 (11.3) | 0.30 |
| Male sex, (%) | 23 (79.3) | 11 (73.3) | 12 (85.7) | 0.65 |
| BMI, kg/m^2^ | 28.6 (5.1) | 28.8 (5.9) | 28.3 (4.2) |  |
| Hypertension, (%) | 16 (55.1) | 7 (47) | 9 (64.3) | 0.4 |
| Diabetes Mellitus, (%) | 3 (10.3) | 2 (13.3) | 1 (7.1) | 0.52 |
| Dyslipidemia, (%) | 8 (27.6) | 4 (26.7) | 4 (28.6) | 1.00 |
| LAVi, ml/m² | 32.1 (27.0, 38.5) | 33.5 (28.4, 39.6) | 30.8 (26.1, 36.9) | 0.28 |
| Paroxysmal AF, n (%) | 22 (75.9) | 11 (73.3) | 11 (78.6) | 0.74 |
| Persistent AF, n (%) | 7 (24.1) | 4 (26.7) | 3 (21.4) | 0.74 |
| Thyroid disease, (%) | 2 (6.9) | 1 (6.7) | 1 (7.1) | 1.00 |
| Coronary artery disease, (%) | 2 (6.9) | 1 (6.7) | 1 (7.1) | 1.00 |

# **Supplementary Table 2. Fold regulations, p-values, and FDRs of all 84 miRNAs.**

| miRNA | Fold Regulation | P-Value | FDR  (Adjusted-P-Value) |
| --- | --- | --- | --- |
| hsa-miR-185-5p | 10,39 | 0,316 | 0,520 |
| hsa-miR-21-5p | 7,79 | 0,236 | 0,451 |
| hsa-miR-125b-5p | 7,04 | 0,620 | 0,733 |
| hsa-miR-22-3p | 5,43 | 0,224 | 0,448 |
| hsa-miR-206 | 5,37 | 0,262 | 0,468 |
| hsa-miR-140-5p | 4,49 | 0,544 | 0,692 |
| hsa-miR-125a-5p | 4,17 | 0,631 | 0,737 |
| hsa-miR-100-5p | 3,92 | 0,780 | 0,830 |
| hsa-miR-30c-5p | 3,9 | 0,110 | 0,420 |
| hsa-let-7f-5p | 3,66 | 0,785 | 0,824 |
| hsa-miR-183-5p | 3,64 | 0,133 | 0,399 |
| hsa-miR-342-3p | 3,62 | 0,049 | 0,294 |
| hsa-miR-195-5p | 2,83 | 0,309 | 0,530 |
| hsa-miR-302b-3p | 2,8 | 0,251 | 0,469 |
| hsa-miR-17-5p | 2,77 | 0,368 | 0,562 |
| hsa-miR-31-5p | 2,73 | 0,084 | 0,372 |
| hsa-miR-208b-3p | 2,58 | 0,062 | 0,326 |
| hsa-miR-142-3p | 2,56 | 0,532 | 0,687 |
| hsa-miR-16-5p | 2,47 | 0,402 | 0,602 |
| hsa-miR-221-3p | 2,44 | 0,220 | 0,450 |
| hsa-miR-424-5p | 2,42 | 0,031 | 0,258 |
| hsa-miR-199a-5p | 2,42 | 0,298 | 0,522 |
| hsa-miR-208a-3p | 2,37 | 0,258 | 0,471 |
| hsa-miR-182-5p | 2,3 | 0,337 | 0,544 |
| hsa-miR-149-5p | 2,24 | 0,477 | 0,657 |
| hsa-miR-155-5p | 2,22 | 0,424 | 0,615 |
| hsa-let-7b-5p | 2,12 | 0,931 | 0,931 |
| hsa-miR-224-5p | 2,11 | 0,212 | 0,469 |
| hsa-miR-214-3p | 1,97 | 0,231 | 0,451 |
| hsa-miR-29b-3p | 1,95 | 0,120 | 0,388 |
| hsa-miR-99a-5p | 1,71 | 0,011 | 0,924 |
| hsa-miR-328-3p | 1,57 | 0,069 | 0,342 |
| hsa-miR-143-3p | 1,52 | 0,523 | 0,686 |
| hsa-miR-378a-3p | 1,41 | 0,047 | 0,331 |
| hsa-miR-25-3p | 1,4 | 0,166 | 0,410 |
| hsa-miR-29c-3p | 1,34 | 0,113 | 0,380 |
| hsa-miR-30d-5p | 1,29 | 0,100 | 0,400 |
| hsa-miR-93-5p | 1,22 | 0,018 | 0,504 |
| hsa-miR-423-3p | 1,22 | 0,044 | 0,336 |
| hsa-miR-130a-3p | 1,2 | 0,602 | 0,733 |
| hsa-miR-365a-3p | 1,16 | 0,048 | 0,310 |
| hsa-let-7d-5p | 1,1 | 0,735 | 0,792 |
| hsa-miR-7-5p | 1,09 | 0,022 | 0,370 |
| hsa-miR-23a-3p | 1,08 | 0,198 | 0,450 |
| hsa-miR-27b-3p | -1 | 0,135 | 0,392 |
| hsa-miR-494-3p | -1,03 | 0,025 | 0,304 |
| hsa-miR-30a-5p | -1,03 | 0,111 | 0,404 |
| hsa-miR-223-3p | -1,09 | 0,216 | 0,466 |
| hsa-miR-30e-5p | -1,14 | 0,095 | 0,398 |
| hsa-miR-98-5p | -1,17 | 0,013 | 0,539 |
| hsa-miR-29a-3p | -1,18 | 0,130 | 0,404 |
| hsa-let-7a-5p | -1,19 | 0,701 | 0,785 |
| hsa-miR-210-3p | -1,21 | 0,875 | 0,896 |
| hsa-miR-107 | -1,26 | 0,456 | 0,638 |
| hsa-miR-181b-5p | -1,27 | 0,346 | 0,548 |
| hsa-let-7c-5p | -1,27 | 0,895 | 0,906 |
| hsa-miR-103a-3p | -1,39 | 0,725 | 0,790 |
| hsa-miR-92a-3p | -1,41 | 0,020 | 0,413 |
| hsa-miR-27a-3p | -1,42 | 0,144 | 0,403 |
| hsa-miR-15b-5p | -1,44 | 0,410 | 0,604 |
| hsa-miR-133a-3p | -1,44 | 0,575 | 0,710 |
| hsa-miR-451a | -1,45 | 0,030 | 0,276 |
| hsa-miR-499a-5p | -1,49 | 0,024 | 0,339 |
| hsa-miR-18b-5p | -1,64 | 0,311 | 0,522 |
| hsa-miR-146a-5p | -1,72 | 0,480 | 0,651 |
| hsa-miR-124-3p | -1,76 | 0,652 | 0,751 |
| hsa-miR-181a-5p | -1,81 | 0,348 | 0,541 |
| hsa-miR-1-3p | -1,84 | 0,056 | 0,314 |
| hsa-miR-24-3p | -1,84 | 0,172 | 0,413 |
| hsa-miR-222-3p | -1,89 | 0,218 | 0,458 |
| hsa-miR-23b-3p | -2,07 | 0,178 | 0,415 |
| hsa-miR-320a-3p | -2,08 | 0,082 | 0,381 |
| hsa-miR-486-5p | -2,13 | 0,029 | 0,305 |
| hsa-let-7e-5p | -2,15 | 0,857 | 0,888 |
| hsa-miR-26b-5p | -2,43 | 0,158 | 0,416 |
| hsa-miR-302a-3p | -2,96 | 0,112 | 0,392 |
| hsa-miR-150-5p | -3,03 | 0,439 | 0,625 |
| hsa-miR-122-5p | -3,15 | 0,666 | 0,756 |
| hsa-miR-144-3p | -3,21 | 0,516 | 0,688 |
| hsa-miR-26a-5p | -3,27 | 0,160 | 0,407 |
| hsa-miR-133b | -5,66 | 0,558 | 0,699 |
| hsa-miR-145-5p | -5,99 | 0,151 | 0,409 |
| hsa-miR-126-3p | -6,18 | 0,617 | 0,740 |
| hsa-miR-10b-5p | -11,6 | 0,709 | 0,783 |

# **Supplementary Table 3. Comparative AUROC values for each miR in predicting AF recurrence after catheter ablation.**

|  | **hsa- miR-486-5p** | **hsa-miR-342-3p** | **hsa-miR-424-5p** | **hsa-miR-10b-5p** |
| --- | --- | --- | --- | --- |
| **AUROC** | 0.68 | 0.78^*^ | 0.76^*^ | 0.96^*,†,‡^ |
| Superscript symbols indicate statistically significant differences between AUROC values as determined by pairwise DeLong tests:  * *p* < 0.05 vs. hsa- miR-486-5p; † *p* < 0.05 vs. hsa-miR-342-3p; ‡ *p* < 0.05 vs. hsa-miR-424-5p. | | | | |

# **Supplementary Table 4. Improvement in model fit after inclusion of individual and combined miRs to the baseline clinical model for predicting AF recurrence after catheter ablation.**

| **Variable** | **Δ(−2LL)** | **ΔR²** | **P-value** |
| --- | --- | --- | --- |
| Model 1 (Reference) |  |  |  |
| Model 1 + hsa-miR-342-3p | 14.403 | 22 | <0.001 |
| Model 1 + hsa-miR-424-5p | 7.645 | 12 | 0.006 |
| Model 1 + hsa-miR-10b-5p) | 50.908 | 63.3 | <0.001 |
| Model 1 + all miRs | 52.535 | 63.5 | <0.001 |
| Model 1 included clinical covariates: male sex, age, body mass index, hypertension, dyslipidemia, diabetes mellitus, left atrial volume index, and paroxysmal AF status. Δ(−2LL) represents the change in −2 log likelihood between the baseline and extended model; ΔR² corresponds to the increase in Nagelkerke R²; and *p*-values derive from the likelihood-ratio test comparing nested logistic regression models. | | | |

# **Supplementary Table 5. ROC Curve analysis of predicted probabilities from logistic regression models based on log2 fold change of candidate miRs.**

| **Model** | **AUC** | **P-value** |
| --- | --- | --- |
| Model 1 (Log2 FC hsa-miR-342-3p) | 0.85 | <0.001 |
| Model 2 (Log2 FC hsa-miR-424-5p) | 0.80 | <0.001 |
| Model 3 (Log2 FC hsa- miR-486-5p) | 0.76 | 0.001 |
| Model 4 (Log2 FC hsa-miR-10b-5p) | 0.98 | <0.001 |
| Models included the following clinical covariates: male sex, age, body mass index, hypertension, dyslipidemia, diabetes mellitus, left atrial volume index, and paroxysmal AF status. | | |

**Supplementary Figure 1. Stepwise analytical workflow for the identification and validation of circulating miRNAs associated with AF recurrence after ablation.**

**Supplementary Figure 2. Stability of endogenous reference controls used for normalization.**
Ct distributions of SNORD38B and SNORD44 (selected using the geNorm algorithm) are shown in patients with AF recurrence and without recurrence, demonstrating comparable expression across groups and supporting their use as stable endogenous references for normalization.

**Supplementary Figure 3. ROC curves comparing predictive models for AF recurrence after ablation.**

Model 1 (red) includes clinical and echocardiographic variables (sex, age, BMI, hypertension, dyslipidemia, diabetes mellitus, and left atrial volume index and paroxysmal AF status).

Model 2 (green) adds hsa-miR-10b-5p to Model 1, and Model 3 (blue) incorporates all candidate miRs.

**Supplementary Figure 4. FOXO signaling pathway targeted by hsa-miR-10b-5p.**

KEGG pathway map showing experimentally validated miR-10b-5p targets (highlighted in yellow), including *FOXO3, PTEN, PDPK1, MAPK1,* and *BCL2L1*.

KEGG pathway map was retrieved and rendered using Pathview (v1.38.0) based on the Kyoto Encyclopedia of Genes and Genomes (KEGG) database.

**Supplementary Figure 5. p53 signaling pathway targeted by hsa-miR-10b-5p.**

Highlighted targets (TP53, MDM2, CDKN1A, CCND2, and BCL2L1) indicate activation of apoptosis and DNA-damage response mechanisms linked to cardiomyocyte stress and fibrosis.

KEGG pathway map was retrieved and rendered using Pathview (v1.38.0) based on the Kyoto Encyclopedia of Genes and Genomes (KEGG) database.

**Supplementary Figure 6. Cell cycle pathway targeted by hsa-miR-10b-5p.**

This pathway shows miR-10b-5p targets (CDKN1A, CDKN2A, CCND2, CDK6, and E2F3) involved in G1/S transition and proliferation control.

KEGG pathway map was retrieved and rendered using Pathview (v1.38.0) based on the Kyoto Encyclopedia of Genes and Genomes (KEGG) database.

**Supplementary Figure 7. Cellular senescence pathway targeted by hsa-miR-10b-5p.**

Key targets (*TP53, CDKN1A, CDKN2A, MDM2,* and *PTEN*) regulate the senescence-associated secretory phenotype (SASP) and oxidative stress response.

KEGG pathway map was retrieved and rendered using Pathview (v1.38.0) based on the Kyoto Encyclopedia of Genes and Genomes (KEGG) database.

**Supplementary Figure 8. Circadian rhythm pathway targeted by hsa-miR-10b-5p.**

Highlighted genes (RORA, NR1D1, CREB1, and PRKAA3) link circadian control of metabolism and redox balance to electrophysiological homeostasis.

KEGG pathway map was retrieved and rendered using Pathview (v1.38.0) based on the Kyoto Encyclopedia of Genes and Genomes (KEGG) database.
