# Supplementary figures and images for: Circulating miR-10b-5p as a candidate biomarker of atrial fibrillation recurrence after catheter ablation: a two-phase translational study
Source: Europace. 2026 Apr 28;28(5):euag097. doi: 10.1093/europace/euag097 (PMC13179786; doi:10.1093/europace/euag097)

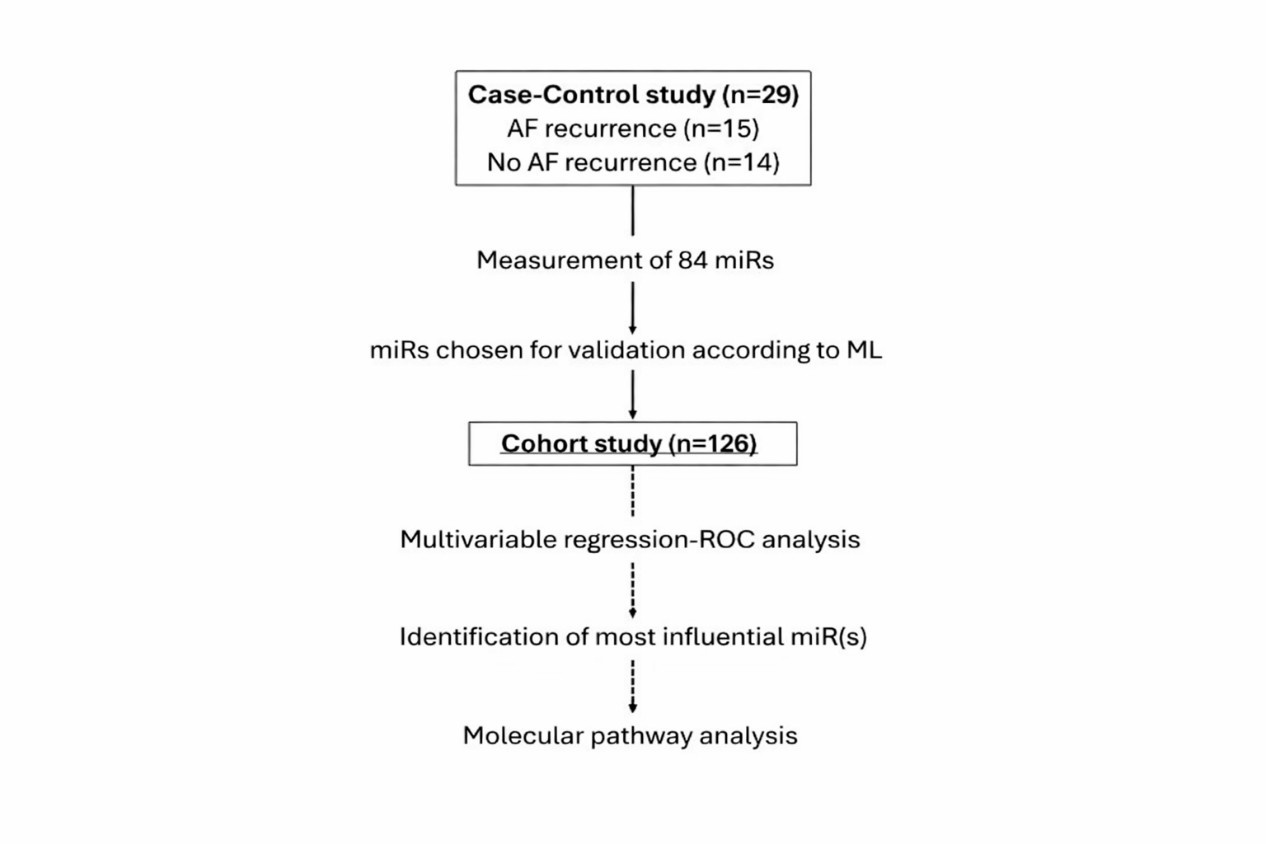

Supplement: euag097_Supplementary_Data [file euag097_supplementary_data.zip › Supplementary Figure 1.jpg]

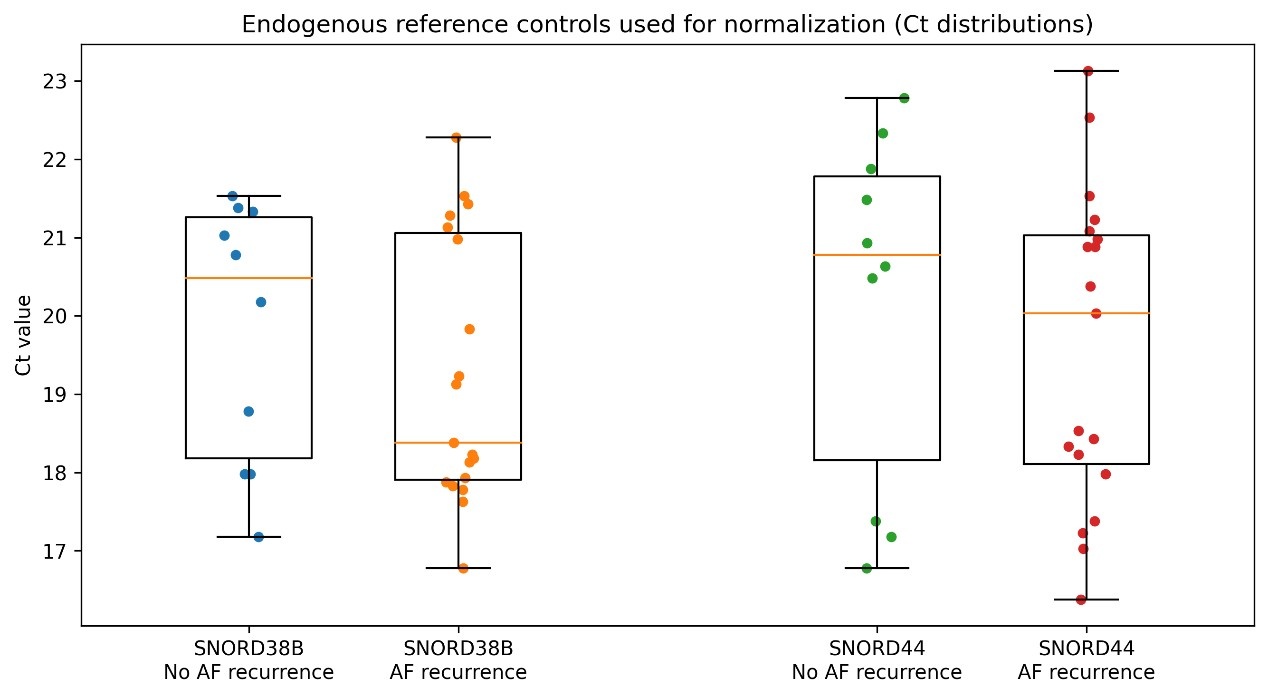

Supplement: euag097_Supplementary_Data [file euag097_supplementary_data.zip › Supplementary Figure 2.jpg]

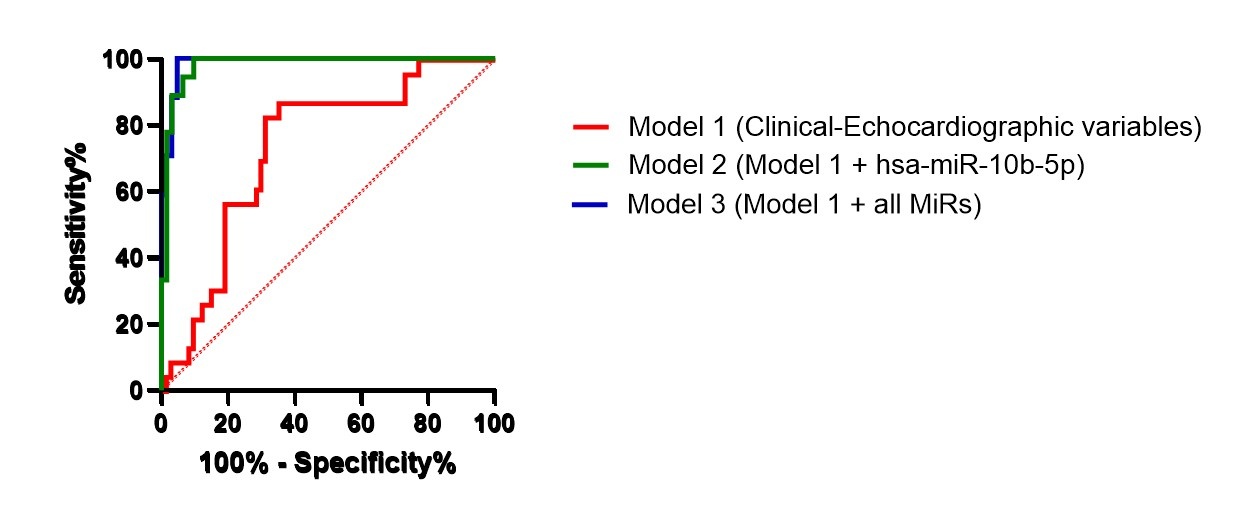

Supplement: euag097_Supplementary_Data [file euag097_supplementary_data.zip › Supplementary Figure 3.jpg]

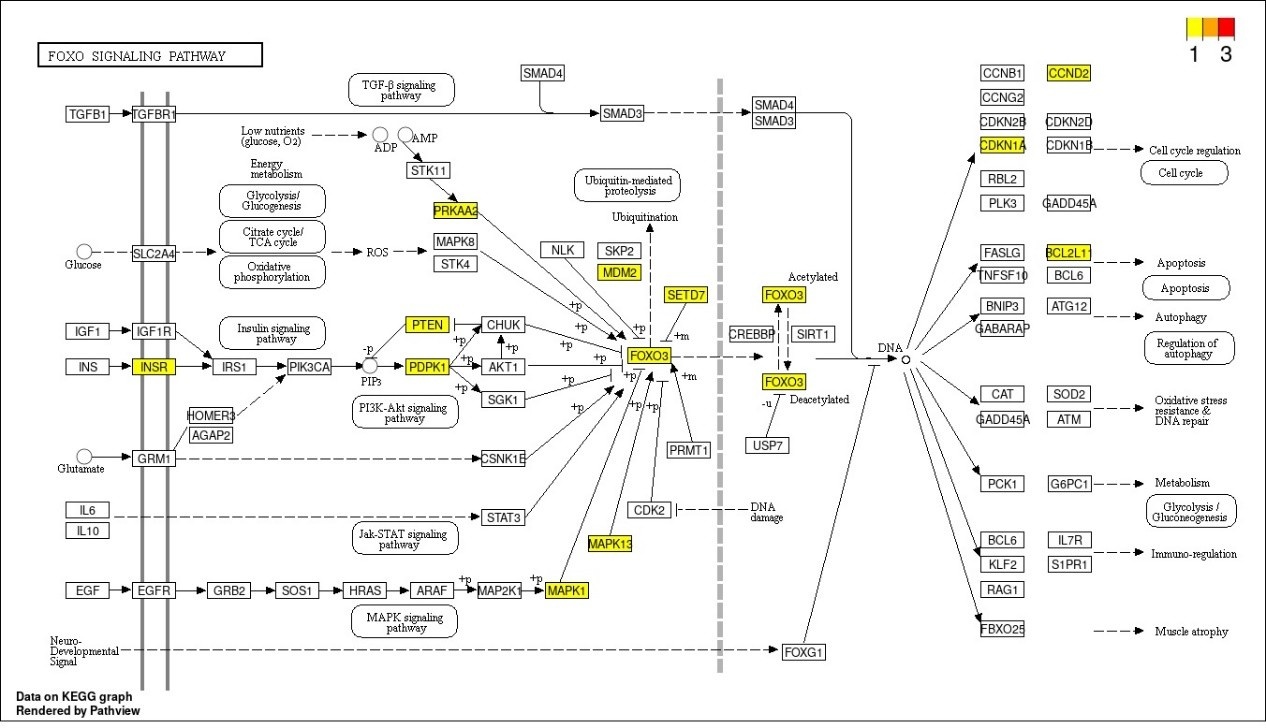

Supplement: euag097_Supplementary_Data [file euag097_supplementary_data.zip › Supplementary Figure 4.jpg]

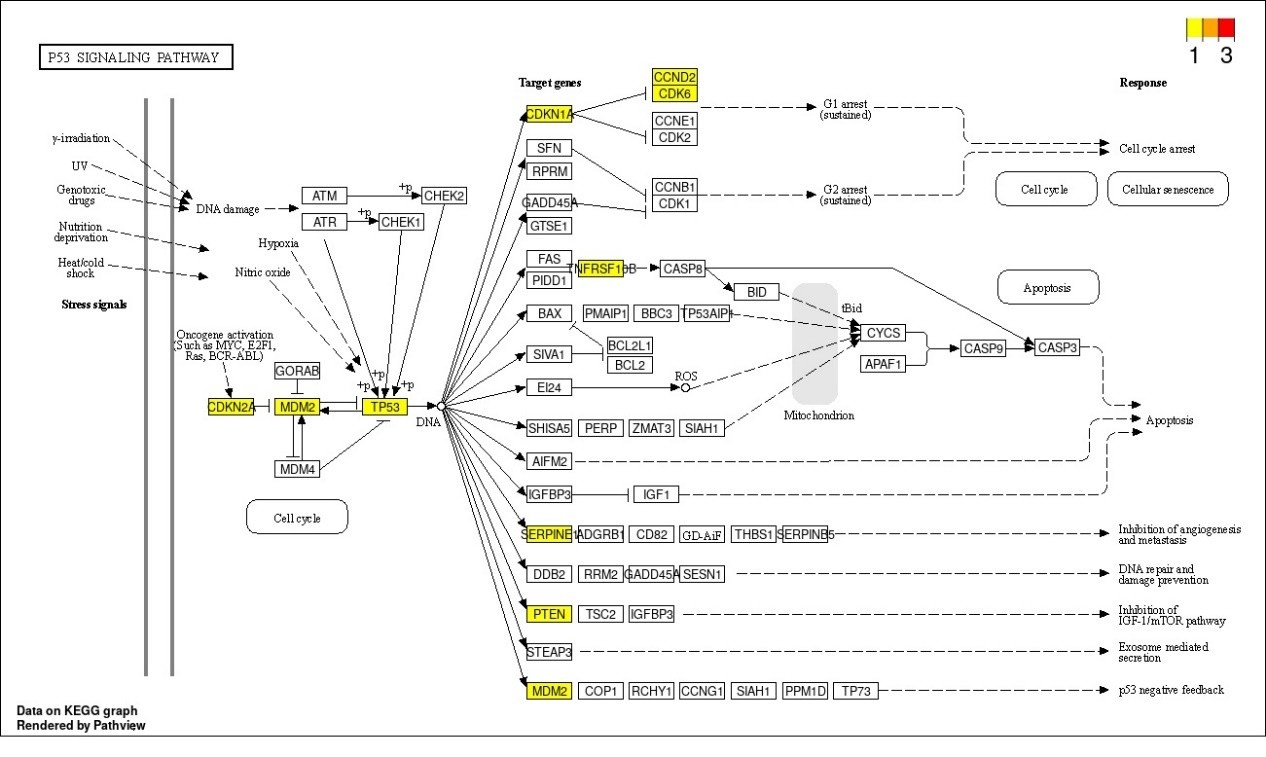

Supplement: euag097_Supplementary_Data [file euag097_supplementary_data.zip › Supplementary Figure 5.jpg]

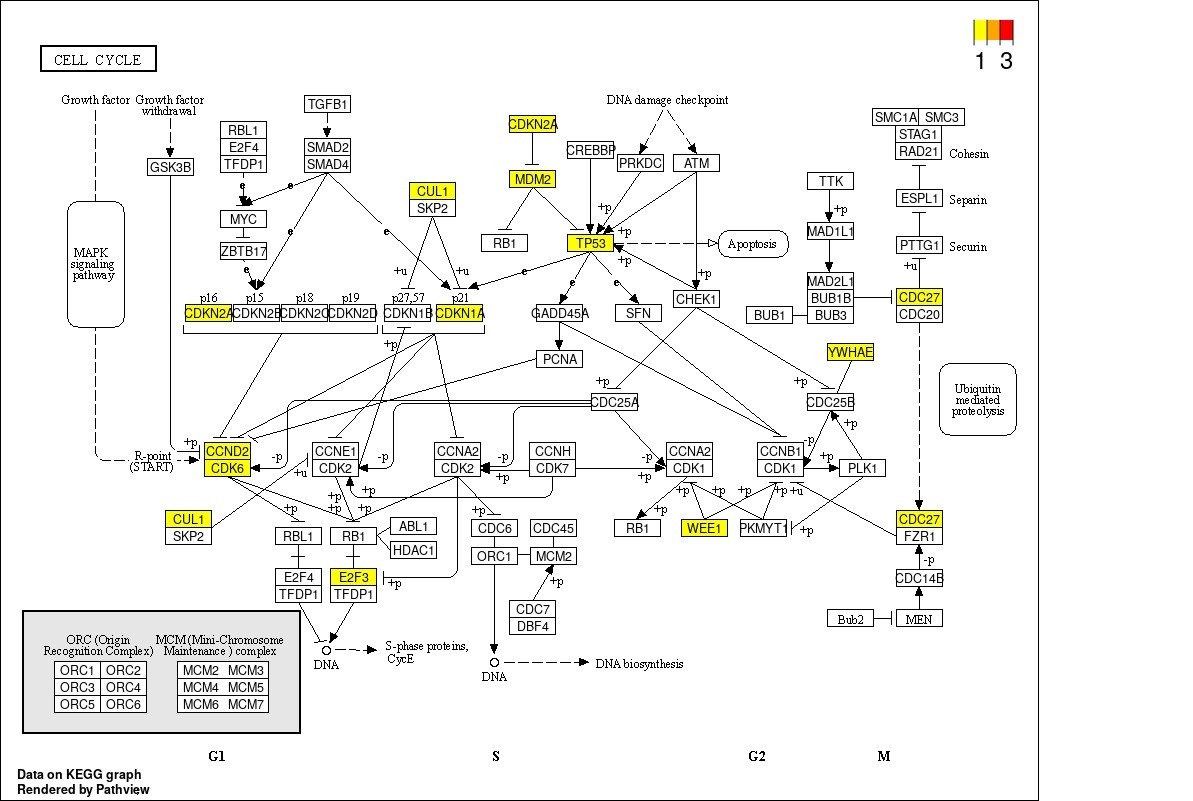

Supplement: euag097_Supplementary_Data [file euag097_supplementary_data.zip › Supplementary Figure 6.jpg]

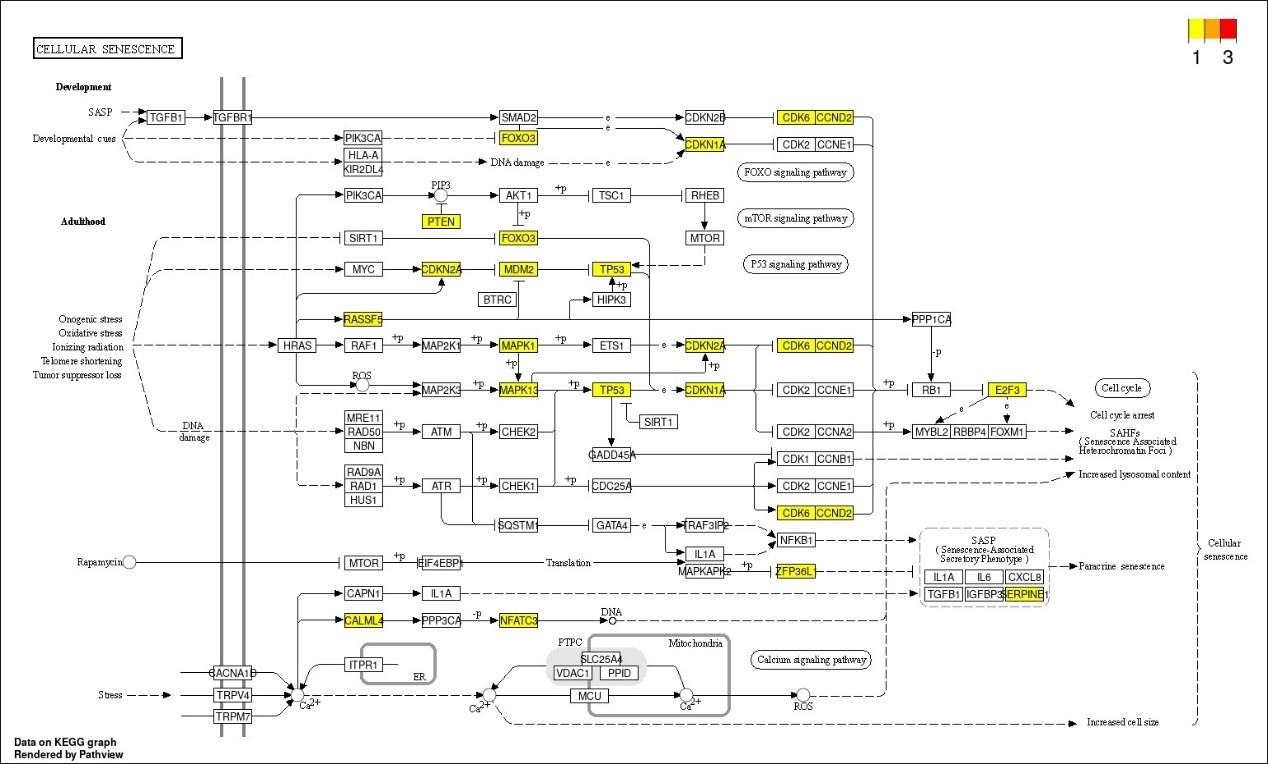

Supplement: euag097_Supplementary_Data [file euag097_supplementary_data.zip › Supplementary Figure 7.jpg]

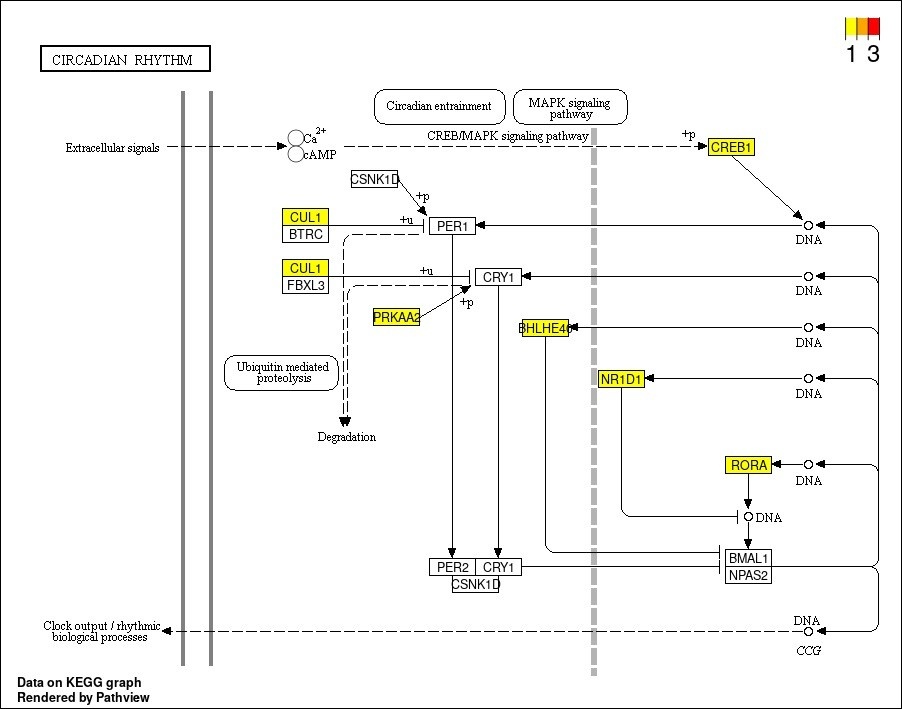

Supplement: euag097_Supplementary_Data [file euag097_supplementary_data.zip › Supplementary Figure 8.jpg]
